# The mid-domain effect of mountainous plants is determined by community life form and family flora on the Loess Plateau of China

**DOI:** 10.1038/s41598-021-90561-4

**Published:** 2021-05-26

**Authors:** Manhou Xu, Rong Du, Xiaoli Li, Xiaohui Yang, Baogui Zhang, Xiuli Yu

**Affiliations:** 1 Institute of Geographical Science, Taiyuan Normal University, Jinzhong, 030619 China; 2 Key Laboratory of Restoration Ecology of Cold Area in Qinghai Province, Northwest Institute of Plateau Biology, Chinese Academy of Sciences, Xining, 810008 China

**Keywords:** Ecology, Ecology

## Abstract

The mid-domain effect (MDE) explains altitudinal patterns of species diversity of mountainous plants at different elevations. However, its application is limited by the species life form and family flora in different layers of plant communities. To verify the MDE hypothesis at the plant community level, we chose a mountain with representative characteristics of the study area in the east of the Loess Plateau, China, such as obvious elevation (from 1324 to 2745 m) and latitude (from 36° 23′ to 39° 03′) gradients and considerable vegetation types (mainly coniferous and broad-leaved forests). We measured the life forms, families, and species diversity indices of tree, shrub, and herb communities along different elevations. We determined that the family numbers of the herb and shrub communities presented unimodal patterns across an altitudinal gradient, and the highest values occurred at intermediate elevations. The importance values of dominant families in the shrub and tree communities presented unimodal patterns, but the lowest values occurred at intermediate elevations. The species diversity indices of the herb, shrub, and tree communities conformed to unimodal change patterns following an altitudinal gradient, but the greatest diversity occurred at high, low, and intermediate elevations, respectively. At higher elevations, forbs and grasses grew well, whereas sedges grew well at lower elevations. Responses of different tree life forms to the altitudinal gradient were greater for evergreen coniferous tree species than for deciduous coniferous and deciduous broad-leaved tree species. We concluded that the MDE hypothesis of species diversity for mountainous plants is influenced greatly by the community life form and family at the plant community level in a temperate semi-arid region of the Loess Plateau, China. This conclusion tested and modified the MDE hypothesis and may be valuable for fueling prediction of biodiversity models and for the comparison with similar studies in arid and semi-arid mountainous regions.

## Introduction

Biodiversity has four components at different scales: landscape diversity, ecosystem diversity, species diversity, and genetic diversity. Species diversity, as an essential component of biodiversity, is the simplest and most effective method to determine community and regional diversity^1–4^. In addition to richness and evenness of species, the distribution variation in species diversity along environmental gradients is a hot topic in biodiversity research. Gradient patterns of species diversity are also the foundation of conservation biology, together with the ecological factors that control these patterns. Species distribution is the outcome of multiple ecological processes that are controlled by species evolution, geographic variation, environmental factors, and biotic interactions^5–8^. The distribution patterns of species diversity are therefore correlated with climate, community productivity, plant evolutionary history, and anthropogenic impact (e.g., deforestation, grazing, and land-use changes)^9–11^. The ecological factors that impact the distribution patterns of species diversity interact with each other and act on species together at a regional scale; thus, they are difficult to distinguish in studies on gradient features of species diversity^12,13^. Taking geographical factors as an example, latitudinal and altitudinal gradients are mixed together to function on species in mountain systems from north to south, and they have become a vital aspect of research on the gradient patterns of species diversity due to their strong relationships with temperature, humidity, and radiation. Moreover, variations in these environmental factors occur 1000 times faster along altitudinal than latitudinal gradients^14,15^. Therefore, the spatial pattern of species diversity along altitudinal gradients has become an increasingly important object for ecologists in mountain research.

In mountains, altitudinal gradients have significant influences on the redistribution of hydrothermal resources (e.g., water availability and temperature), which indirectly affects the species composition and community structure of the plant community^16–18^. Therefore, variations in the species diversity of a plant community form an interesting subject for ecologists in response to altitudinal gradients across mountains^19–21^. However, research has yielded conflicting results on species diversity variation patterns in mountainous plant communities along altitudinal gradients. The correlations between species diversity and elevation can be divided into five groups: negative correlation^22^, positive correlation^23^, or no correlation^24^, and the highest^25^ or lowest^26^ values that can occur at medium elevations. In recent years, considerable research conducted on various mountains has verified the conclusion that the species diversity of mountainous plant communities reaches a maximum at intermediate elevations, which supports the mid-domain effect (MDE) hypothesis^27^.

The MDE hypothesis is another vital mechanism that elucidates species diversity patterns in addition to the environmental gradient, area, heterogeneity, and disturbance; it has been proposed by ecologists based on species overlap and border restriction in distribution along altitudinal gradients^28–30^. According to the MDE hypothesis, a species is continuously distributed across its distribution range, and the ranges of different species overlap; within a domain, owing to the restriction of borders on species distribution, the ranges of different species have smaller overlapping degrees at the border region and greater overlap in the central region, which results in more species in the central region and larger species diversity, namely, the unimodal distribution pattern of species diversity^31^. Nevertheless, at different scales, species diversity has disparate distribution patterns along altitudinal gradients and their formative factors differ greatly, so scales may strongly affect the MDE hypothesis. These scales include those along environmental gradients, especially those in different layers of plant communities with species that have various life forms and belong to different families^32^. In conclusion, the MDE hypothesis of mountainous plant community needs to be further tested along altitudinal gradients and the influential factors of the hypothesis should be explored at the plant community level related to different layers of communities, particularly those factors on life form and family flora of plant species.

In order to obtain a better validation of the MDE hypothesis, measurements of species diversity are primarily conducted at three spatial scales known as α-, β-, and γ-diversity^33^. Among these types of diversity, α-diversity (within-habitat diversity) principally focuses on species number in a homogeneous habitat at local scales. At this scale, the principle factors that affect diversity are ecological niche and interspecific interaction. These factors are closely related to environmental resources and vegetation community structures, and thus α-diversity can reflect variations of plant species along environmental gradients^34^. As a result, α-diversity is preferentially used to explain the MDE hypothesis^33,35^. α-diversity elucidates two driving factors of species distribution in all kinds of environments. One points to the number of species, namely, the species richness, which can be further described as Simpson index and Shannon index; the other shows the evenness of species with the Pielou index being a common index^1^. These indices (Simpson, Shannon, and Pielou indices) indicate changes of species number and distribution along environmental gradients in diverse layers of plant communities, and thus are used to test the MDE hypothesis.

In arid and semiarid mountainous regions, some fragile ecosystems where plant diversity exhibits an obvious MDE are threatened most severely by hydrothermal factors. This can be seen on the Loess Plateau^36,37^. The plateau has the most severe soil erosion and the most fragile ecological environment in China, and possibly the world, due to its semi-arid climate, frequent droughts, low precipitation, and sparse vegetation. A warm-temperate and mid-temperate zone occur from south to north across the plateau, and a semi-arid and arid region occur from east to west; thus, although it is low in vegetation coverage, there are abundant types of vegetation on the plateau that exhibit typically community structure characteristics along diverse elevations, including grassland, bush, broad-leaved forest, mixed coniferous broad-leaved forest, coniferous forest, and subalpine meadow^38,39^. These types of vegetation have distinct layers and can be divided into various communities with species possessing different life forms and belonging to different families. Thus, the plateau is an ideal region to verify the MDE hypothesis at the plant community level. To this purpose, the Lvliang Mountain system in the eastern part of the plateau, with obvious elevation gradients and vegetation structures, was selected as a model mountain and divided into various belts with different elevation gradients. In each belt, forest ecosystems were separated into three communities: the tree community, shrub community, and herb community. Plant species with different life forms and families were also determined in each community. Surveys on vegetation characteristics were first conducted in each community, and then species diversity together with its altitudinal patterns were correspondingly calculated and analyzed across different elevations. The influential factors were also determined with respect to the MDE hypothesis at the plant community level, namely the species life form and family flora in diverse layers of plant communities. Through this research, we made a core hypothesis that the MDE hypothesis of species diversity for mountainous plants is influenced greatly by the community life form and family flora at the plant community level and that the hydrothermal distribution status affected by latitude determined the altitudinal distribution patterns of plant community diversity on the Loess Plateau of China.

## Methods

### Study area

**Figure 1:**
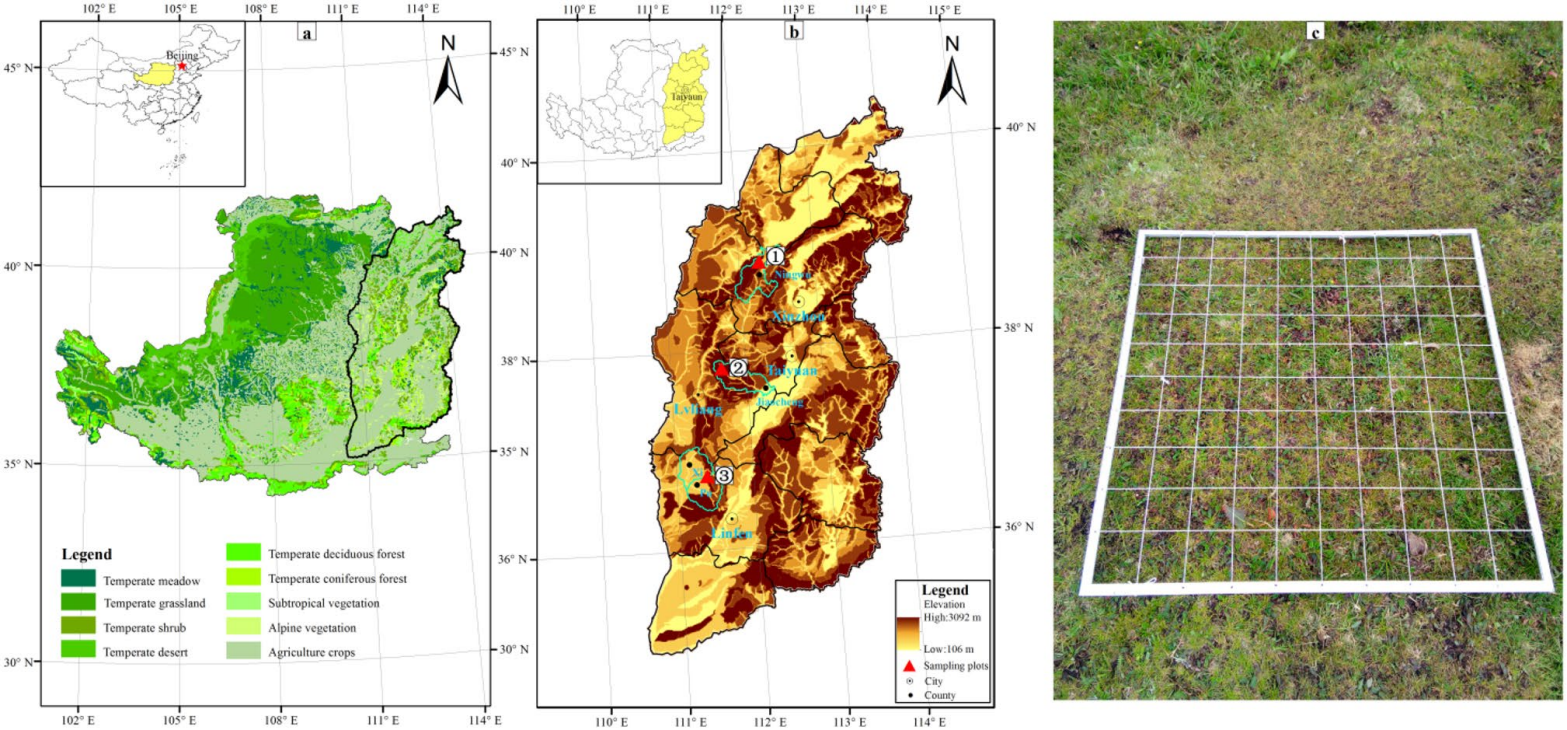
Maps of the study area indicating vegetation types on the Loess Plateau (**a**), the topography in Shanxi Province (**b**), and aluminum alloy frame (**c**). Along an altitudinal gradient, communities across 9, 8, and 4 elevations were observed at Guancen Mountain (①), Guandi Mountain (②), and Wulu Mountain (③), respectively. In the interior of the frame, 100 grids were separated to accurately measure plant frequency and coverage. (**a**) and (**b**) were created by using ArcMap 10.2 (https://desktop.arcgis.com/zh-cn/arcmap/) in the ArcGIS software with the data on spatial distribution of vegetation types and geomorphic types being all derived from the Resource and Environmental Science and Data Center (http://www.resdc.cn/). The boundary map of the Loess Plateau in (**a**) and cities of the Shanxi Province in (**b**) were generated by the data from the National Earth System Science Data Center (http://gre.geodata.cn) and the Resource and Environmental Science and Data Center (http://www.resdc.cn/), respectively.

The Lvliang Mountain system belongs to an eastern mountain of the Loess Plateau (Fig. 1a) with a length of about 400 km from south to north and a width of about 50 km from east to west, including Guancen Mountain in the northern section, Guandi Mountain in the middle section, and Wulu Mountain in the southern section (mean latitudes: 38.5° N, 37.5° N, and 36° N, respectively) (Fig. 1b).

Guancen Mountain is located at Ningwu County in Xinzhou City with geographical coordinates of 38° 57′–39° 03′ (N latitude) and 112° 36′–112° 37′ (E longitude). The sample plots across this mountain have elevations from 1740 to 2745 m, an annual mean air temperature of 6–7 °C, average annual precipitation of 450–500 mm, and a frost-free season of 90–120 days. From low to high elevations, the soil types are cinnamon soil, brown soil, and subalpine meadow soil, and the vegetation includes bush, mixed coniferous broad-leaved forest, coniferous forest, and subalpine meadow.

Guandi Mountain is located at Jiaocheng County in Lvliang City with geographical coordinates of 37° 20′–38° 20′ (N latitude) and 110° 18′–111° 18′ (E longitude). The sample plots across this mountain have elevations from 1800 to 2690 m, an annual mean air temperature of 3–4 °C, average annual precipitation of 600–800 mm, and a frost-free season of 100–130 days. From low to high elevations, the soil types are also cinnamon soil, brown soil, and subalpine meadow soil, and the vegetation includes broad-leaved forest, mixed coniferous broad-leaved forest, coniferous forest, and subalpine meadow.

Wulu Mountain is located at a junction between Pu and Xi counties in Linfen City, with geographical coordinates of 36° 23′–36° 38′ (N latitude) and 111° 2′–111° 18′ in (E longitude). The sample plots across this mountain have elevations from 1324 to 1586 m, an annual mean air temperature of 8–9 °C, average annual precipitation of 500–560 mm, and a frost-free season of 150–180 days. From low to high elevations, the soil types are cinnamon soil and brown soil, and the vegetation includes bush and broad-leaved forest.

### Arrangement of survey plots

The Lvliang Mountains have apparent elevations, high vegetation coverage, and diverse vegetation types. Due to these factors, these mountains are an ideal region to determine the altitudinal patterns of species diversity of various plant communities. In previous studies conducted in July of 2015^35^, we determined the plant community characteristics across 12 elevation points along an altitudinal gradient. On this basis, from July to August in 2017, we carried out new selections and designed multiple survey plots on this mountain for a period of one month. These survey plots covered various plant communities with species that had different life forms and belonged to families in diverse layers.

For each mountain in the vertical direction, survey plots were established according to an elevation gradient to investigate the species diversity of plant communities. The plant communities were divided into three layers: tree, shrub, and herb layers. In total, 21 elevation points were selected along an elevation gradient from the three mountains: 1324, 1370, 1459, 1586, 1740, 1800, 1892, 1900, 1950, 2001, 2179, 2222, 2270, 2395, 2460, 2571, 2610, 2666, 2675, 2690, and 2745 m. The difference between the highest and the lowest elevations was 1421 m. Among these elevation points, the tree community covered 13 points: 1459, 1586, 1740, 1892, 1900, 1950, 2001, 2179, 2222, 2270, 2395, 2571, and 2610 m; the shrub community covered 6 points: 1324, 1370, 1586, 1800, 1950 and 2675 m; and the herb community covered 14 points: 1324, 1459, 1586, 1900, 2001, 2179, 2222, 2395, 2460, 2571, 2666, 2675, 2690, and 2745 m.

At each elevation point, quadrats with different areas were designed for investigation based on the type of plant community present. These quadrats were divided into sub-quadrats in order to make the investigation easier. The number and area of quadrats in the tree community were 2 and 1000 m^2^, respectively, and the sampling area was 2000 m^2^ in total. These quadrats were divided into 20 sub-quadrats with areas of 10 m × 10 m. The quadrat number and area in the shrub community were 4 and 400 m^2^, respectively, and the total sampling area was 1600 m^2^. These quadrats were divided into 64 sub-quadrats with areas of 5 m × 5 m. In the herb community, 20 quadrats were investigated, each with an area of 1 m^2^. These quadrats were divided into 80 sub-quadrats with area of 0.5 m × 0.5 m based on the total sampling area of 20 m^2^. Along the 21 elevation points in all of the mountains, the total number of surveyed sub-quadrats were 260, 384, and 1120 in the tree, shrub, and herb communities, respectively. During data processing, different individuals of the same plant species were merged from all of the sub-quadrats of each community type along each elevation point and then characteristic indices of all of the individuals were averaged for each plant species.

### Measurement of plant growth characteristics

The methods adopted in the characteristic measurements of the plant communities here were in accordance with those in previous research^35^. The measurement indices for the tree species included individual amount, diameter at breast height, basal diameter, plant height, and crown breadth; those for the shrub species included individual amount, plant height, and crown breadth; and those for the herb species included individual amount and plant height (data in Supplementary Table S1). Basing on these plant growth indices, plant species diversity (α-diversity) was calculated as follows (see “Data analysis”).

The density of tree, shrub, and herb species was calculated by the ratio between the plant individual amount and the corresponding quadrat areas. The height of tree species was estimated with visual observation. The frequency and coverage of herb species were calculated using a measurement tool of homemade aluminum frame with a size of 1 m × 1 m. The interior of this tool was separated into 100 grids, each 10 cm × 10 cm in size (Fig. 1c). For each grid, we recorded whether a grid touched bare ground, litter, or plant species. Frequency was calculated by the number of grids in which a plant appeared divided by 100. Coverage was calculated by the number of grids touching plants divided by 100.

During the measurements, tree species with a height of less than 5 m were classified into the shrub layer, and seedlings of woody plants were considered herb layer if their heights were less than 0.2 m. Definite life forms of various species were confirmed using the Flora of China (http://foc.eflora.cn/). The specific measurement methods have been described in detail^35^.

### Data analysis

With the purpose of further verifying the MDE hypothesis, α-diversity indices focused on the local and within-community vegetation characteristics were employed in this study to analyze patterns of species diversity along an altitudinal gradient in the tree, shrub, and herb communities of the Lvliang Mountains, including the Simpson index, Shannon index, and Pielou index^40,41^. These indices were calculated according to the following formulas^33,35,42^ in Microsoft Office Excel 2010.

Importance value is a vital index used to calculate and assess species diversity. It demonstrates the relative significance of species in plant communities^26,35^. However, it was calculated differently for different plant functional types. According to the diverse plant growth indices we measured, importance values were calculated according to plant height, density, and coverage for each tree and shrub species (Eq. 1), and according to plant height, density, frequency, and coverage for each herb species (Eq. 2). The Simpson, Shannon, and Pielou indices were then calculated by Eqs. (3), (4), and (5) based on the relative importance value (Eq. 6), respectively. The growth index of frequency was not obtained for tree and shrub species as their quadrat sampling areas were too large, while the index was easily obtained for herb species using the homemade aluminum frame. We therefore adopted different formulas for tree/shrub communities and for herb communities in order to explore the actual species diversity in different plant communities^33,35,66^.

1 $$IV_{{{\text{Arbor }}\,{\text{and }}\,{\text{shrub}}}} = \frac{rd + rh + rc}{3},$$

2 $$IV_{{{\text{Herb}}}} = \frac{rd + rh + rf + rc}{4},$$

3 $$H^{\prime} = 1 - \sum\limits_{i = 1}^{S} {p_{i}^{2} } ,$$

4 $$H = - \sum\limits_{i = 1}^{S} {p_{i}^{{}} } \ln \left( {p_{i} } \right),$$

5 $$E = \frac{H}{\ln \left( S \right)},\,{\text{and}}$$

6 $$p_{i} = \frac{{IV_{i} }}{{IV_{total} }}.$$

In these equations, *IV* is the importance value, *rd* is the relative density, *rh* is the relative height, *rf* is the relative frequency, and *rc* is the relative coverage; *H′* is the Simpson index, *H* is the Shannon index, and *E* is the Pielou index; and *i* and *S* represent plant species *i* and the total number of plant species present in the frames of the surveyed plots, respectively. In Eqs. (1) and (2), the *rd*, *rh*, *rf*, and *rc* were calculated separately by the ratio between that value of a certain plant species and the total value of all plant species (Eq. 7).

7 $$rd = \frac{{{\text{Density}}_{i} }}{{{\text{Density}}_{total} }},rh = \frac{{{\text{Height}}_{i} }}{{{\text{Height}}_{total} }},rf = \frac{{{\text{Frequency}}_{i} }}{{{\text{Frequency}}_{total} }},{\text{and}}\,rc = \frac{{{\text{Coverage}}_{i} }}{{{\text{Coverage}}_{total} }}.$$

Finally, normal function fittings were conducted on the relationships between species diversity and elevation in the herb, shrub, and tree communities. The conventional functions selected in this study were the exponential function, linear function, logarithmic function, quadratic polynomial function, and power function. By fitting these relationships, the optimal function was determined from the selected functions with the largest determination coefficient (*R*^2^) and the smallest significance test (*P* value). Using the optimal function, a trend of species diversity together with its turning point was clearly revealed along an altitudinal gradient. Meanwhile, the corresponding determination coefficients of these functions indicated the variation ratios of species diversity caused by the altitudinal gradient.

## Results

### Altitudinal patterns of the herb community

#### Variations in herb families with elevations

**Figure 2:**
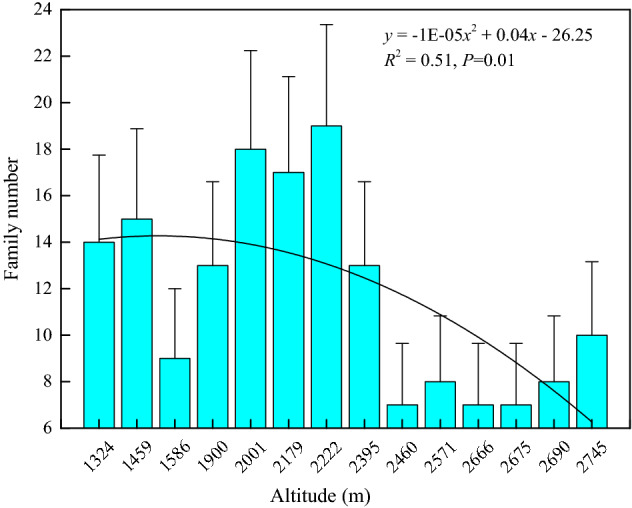
Variation in herb family number with an altitudinal gradient. The family number of the herb community was counted at each elevation, and curve fittings were conducted on the variation in family number with an altitudinal gradient. Data for the family number were obtained from 80 sub-quadrats at each elevation.

At elevation points of 1324, 1459, 1586, 1900, 2179, 2222, 2395, 2571, 2675, and 2745 m, the dominant herb families were all Cyperaceae with a maximum importance value of 42.37%. At 2001 m, Rosaceae was the dominant family with a maximum importance value of 13.24%. At 2460 m, Saxifragaceae was dominant with 19.93% as its maximum importance value. At 2666 m and 2690 m, the dominant families were all Polygonaceae with the mean of maximum importance values being 19.96%. Hence, Cyperaceae was the dominant family in all of the herb communities. The family number increased initially and then decreased with increasing elevations (*P* < 0.01), showing a unimodal change pattern with high values at central elevations (Fig. 2). This showed that the number of families in the herb community reached the maximum at an intermediate elevation.

#### Variations in herb life forms with elevations

**Figure 3:**
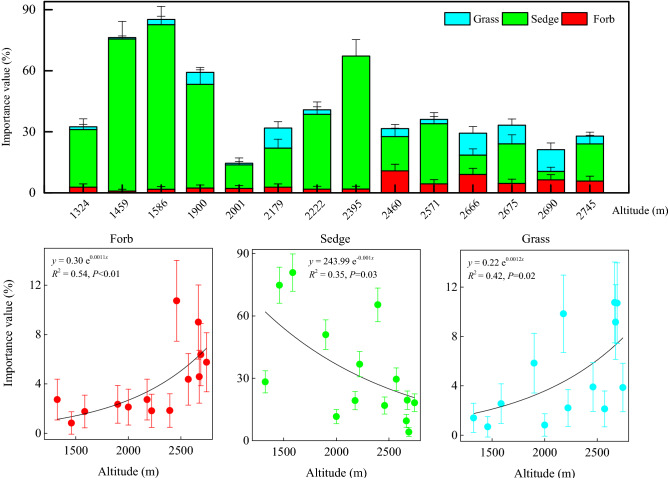
Distribution and variation of the importance values of grasses, sedges, and forbs at different elevations. The grass, sedge, and forb species belonged to Gramineae, Cyperaceae, and other families, respectively. At each elevation, species of the herb community were divided into these three life forms. Their importance values were used in regression analyses with the altitudinal gradient. Data for the importance values of grasses, sedges, and forbs were averaged from 80 sub-quadrats at each elevation.

To compare importance values of diverse life forms, grasses, sedges, and forbs were separated within the herb community (Fig. 3). The importance value of sedges was greater than that of grasses and forbs at different elevations except for at 2690 m. The importance value of the sedges decreased with increasing elevations (*P* < 0.05), while the importance values of the grasses and forbs increased with increasing elevations (*P* < 0.05) and the most significant correlation (*P* < 0.01) occurred between forbs and elevations. The importance values of sedges, grasses, and forbs exhibited significant exponential relationships with elevations (*P* < 0.05); the coefficients of determination were ranked from lowest to highest as sedges, grasses, and forbs. It was concluded that altitudinal gradients have greater effects on forbs, and that more forbs and grasses are distributed at higher elevations, whereas sedges tend to grow at lower elevations.

#### Variations in herb species diversity indices with elevations

In the herb community, species diversity relationships were fitted to an altitudinal gradient using a quadratic polynomial function that explained an average of 37.24% of the variation in species diversity (Table 1). For the Simpson index, all of the functional relationships were significant with respect to the altitudinal gradient (*P* < 0.05), and the highest *R*^2^ value was also associated with a quadratic polynomial function, which indicated that 35.49% of the variation in the Simpson index was caused by the altitudinal gradient. For the Shannon index, all of the functional relationships were non-significant (*P* > 0.05) and the highest *R*^2^ value was associated with a quadratic polynomial function, which indicated that 21.59% of the variation in the Shannon index was caused by the altitudinal gradient. For the Pielou index, all of the functional relationships reached the most significant level (*P* < 0.01) and a quadratic polynomial function resulted in the highest *R*^2^ value in these relationships; according to this function, 54.65% of the variation in the Pielou index was caused by the altitudinal gradient. An increase in elevations resulted in an increase in the Simpson, Shannon, and Pielou indices, illustrating a distribution pattern of greater species diversity at higher elevations, where fewer herb species occurred but were evenly and steadily distributed.

**Table 1:** Various functional relationships between species diversity and elevations. The α-Diversity indices used in this study were the Simpson index, Shannon index, and Pielou index. Data not presented in parentheses are the coefficients of determination of the equations, and data in parentheses are the *P* values of the significance testing of the equations. The numbers of data points are 80, 64, and 20 from sub-quadrats for the herb, shrub, and tree communities, respectively.

Plant type	Diversity index	Exponential function	Linear function	Logarithmic function	Quadratic polynomial function	Power function
Herb	Simpson	0.34 (0.03)	0.35 (0.03)	0.33 (0.03)	0.35 (0.02)	0.33 (0.03)
	Shannon	0.19 (0.32)	0.19 (0.12)	0.19 (0.12)	0.22 (0.09)	0.21 (0.10)
	Pielou	0.49 (0.01)	0.51 (< 0.01)	0.48 (0.01)	0.55 (< 0.01)	0.46 (0.01)
Shrub	Simpson	0.72 (0.05)	0.63 (0.06)	0.57 (0.08)	0.73 (0.14)	0.64 (0.06)
	Shannon	0.64 (0.06)	0.50 (0.12)	0.42 (0.17)	0.69 (0.17)	0.55 (0.09)
	Pielou	0.42 (0.16)	0.44 (0.15)	0.47 (0.13)	0.52 (0.33)	0.45 (0.14)
Tree	Simpson	< 0.01 (0.83)	0.01 (0.75)	< 0.01 (0.90)	0.42 (0.07)	< 0.01 (0.98)
	Shannon	0.06 (0.41)	0.05 (0.46)	0.03 (0.58)	0.40 (0.08)	0.04 (0.52)
	Pielou	0.65 (< 0.01)	0.66 (< 0.01)	0.71 (< 0.01)	0.80 (< 0.01)	0.70 (< 0.01)

### Altitudinal patterns of the shrub community

#### Variations in shrub families with elevations

**Figure 4:**
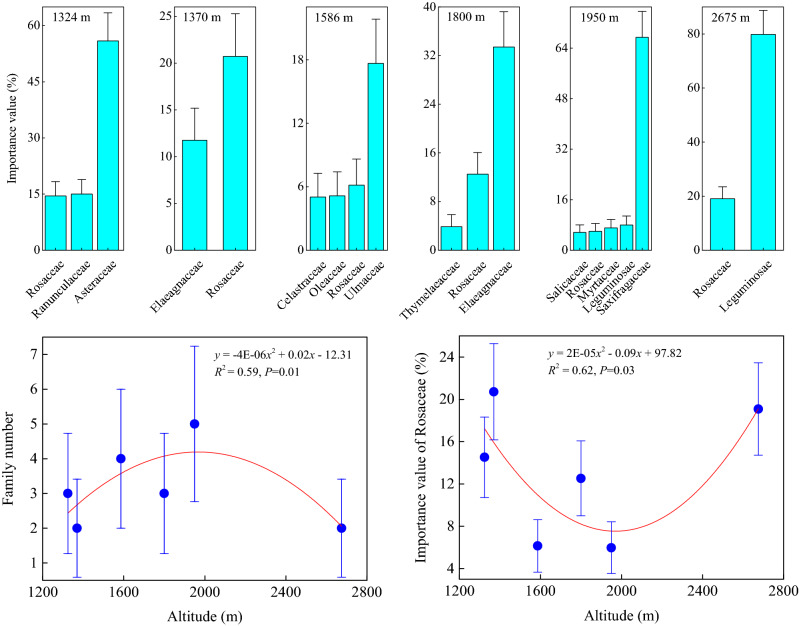
Distribution and variation of importance values and numbers of shrub families at different elevations. There were 6 elevations for the shrub community. At each elevation, the importance values of the shrub families were arranged in ascending order to obtain the dominant family. Data for each shrub family were averaged from 64 sub-quadrats at each elevation. Finally, regression analyses were carried out on relationships between the family number of the shrub community and the importance value of the dominant family (Rosaceae) along the altitudinal gradient.

Relative to the herb community, fewer families in the shrub community were distributed at different elevations, and the variation range was between 2 and 5 families (Fig. 4). The family number exhibited the most significant changes with altitudinal gradient in the shrub community (*P* < 0.01), demonstrating a unimodal pattern with a peak value at intermediate elevations, i.e. the family number of the shrub community had the greatest distribution at an intermediate elevation.

Across the 6 elevations along the altitudinal gradient of the shrub community, the families with the highest importance value were not the same. From the lowest to highest elevations, these families were Asteraceae (55.92%), Rosaceae (20.72%), Ulmaceae (17.65%), Elaeagnaceae (33.44%), Saxifragaceae (67.36%), and Leguminosae (79.83%) (Fig. 4). Rosaceae existed at various elevations with a mean importance value of 13.17% and was thus considered a dominant family in all the shrub communities. The importance value of Rosaceae decreased initially and then increased with increasing elevations (*P* < 0.05), exhibiting a unimodal change pattern with a small value at intermediate elevations, i.e., the importance value of the dominant family in the shrub community was the lowest at an intermediate elevation.

#### Variations in shrub species diversity indices with elevations

The fitting of functional relationships between species diversity indices and elevations in the shrub community indicated that a quadratic polynomial was the best function, though it was not significant (*P* > 0.05, Table 1). Based on this function, the amount of variation in species diversity explained by the altitudinal gradient was higher than that explained by other conventional functions: 73.48% for the Simpson index, 69.2% for the Shannon index, and 51.89% for the Pielou index. Therefore, the relationships between shrub species diversity and elevations was in accordance with the quadratic polynomial functions, and the amount of variation explained by the altitudinal gradient reached 64.86% for the different species diversity indices. Species diversity indices tended to decrease with increasing elevations (*P* > 0.05), indicating that most shrub species are primarily distributed at lower elevations, where species are more balanced and stabilized and thus produce a distribution pattern of greater species diversity.

### Altitudinal patterns of the tree community

#### Variations in tree families with elevations

**Figure 5:**
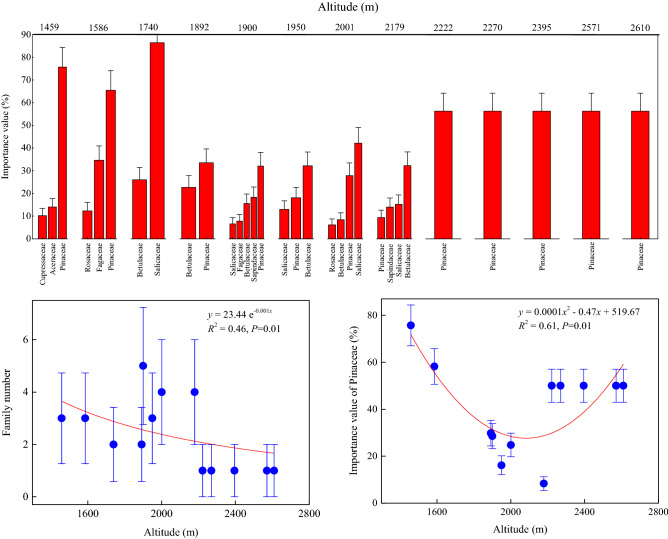
Distribution and variation of the importance values and numbers of tree families at different elevations. Thirteen elevations were observed for the tree community. At each elevation, the importance values of the tree families were arranged in ascending order to obtain the dominant family. Data for each tree family were averaged from 20 sub-quadrats at each elevation. Regression analyses were performed on the relationships between the family number of the tree community and the importance value of the dominant family (Pinaceae) along the altitudinal gradient.

Similar to the shrub community, the tree community also had a smaller distribution at different elevations, with a variation range between 1 and 5 families (Fig. 5). Changes in the exponential function of the family number reached a significant level with an altitudinal gradient in the tree community (*P* < 0.05), assuming a decreasing pattern. That is to say, the family number of the tree community has a maximum distribution at lower elevations.

Across the 13 elevations along the altitudinal gradient of the tree community, the highest importance values were obtained for Pinaceae, Salicaceae, and Betulaceae (Fig. 5). For Pinaceae, maximum importance values with an average of 49.14% occurred at 9 elevations. At intermediate elevations from 1950 to 2179 m, Salicaceae and Betulaceae species dominated, whereas Pinaceae species only appeared at higher elevations exceeding 2222 m, indicating a transformation of the tree community from a mixed coniferous and broad-leaved forest to a pure coniferous forest. Therefore, Pinaceae was a dominant family in all of the tree communities and was distributed across all of the elevations except 1740 m (the pure broad-leaved forest). With increasing elevation, the importance value of Pinaceae initially decreased and then increased (*P* < 0.05), indicating a unimodal change pattern with a small value at intermediate elevations, i.e., the importance value of the dominant tree family is minimal at intermediate elevations.

#### Variations in tree life forms with elevations

According to life forms, tree species were divided into deciduous broad-leaved trees, deciduous coniferous trees, and evergreen coniferous trees (Table 2). Deciduous broad-leaved trees were mainly distributed at medium and low elevations of less than 2179 m and their importance values showed a non-significant tendency with increasing elevations (*R*^2^ < 0.1, *P* > 0.05). Deciduous coniferous trees were mainly distributed at intermediate and high elevations that exceeded 1892 m and their importance values tended to increase with increasing elevations (*R*^2^ = 0.3, *P* > 0.05). Evergreen coniferous trees were distributed across all of the elevations except 1740 m. Their importance values tended to decrease initially and then to increase following an increase in elevation (*R*^2^ = 0.4, *P* > 0.05). Hence, the responses of the importance values of various tree species to the altitudinal gradient were successively greater for evergreen coniferous trees than for deciduous coniferous trees, followed by deciduous broad-leaved trees. It was concluded that the effects of elevation on the importance values of coniferous trees are greater than those of broad-leaved trees, and the effects of elevation on the importance values of evergreen trees are greater than those of deciduous trees.

**Table 2:** Distributions of importance values of various tree species at different elevations. Thirteen elevations represent the tree community. Tree species are divided into deciduous broad-leaved trees, deciduous coniferous trees, and evergreen coniferous trees. “—” indicates no value at that elevation. At 1740 m, there was only one tree species, a deciduous broad-leaved tree. Data for different tree species were obtained from 20 sub-quadrats at each elevation.

Altitude (m)	Importance value (%)		
	Deciduous broad-leaved trees	Deciduous coniferous trees	Evergreen coniferous trees
1459	14.05	–	42.98
1586	20.89	–	58.21
1740	50.00	–	–
1892	20.21	11.10	48.49
1900	10.73	11.80	45.29
1950	17.22	35.36	6.50
2001	16.82	16.16	33.40
2179	20.83	4.49	12.21
2222	–	59.21	40.786
2270	–	41.07	58.93
2395	–	32.20	67.80
2571	–	39.35	60.65
2610	–	32.55	67.45

#### Variations in tree species diversity indices with elevations

The fitting of regular functions indicated that quadratic polynomial functions also existed in correlations between species diversity indices and elevations in the tree community (Table 1). In these correlations, the Pielou index reached the most significant level with respect to the altitudinal gradient (*P* < 0.01). The amount of variation in species diversity indices explained by the altitudinal gradient was higher for the quadratic polynomial functions than for the other regular functions, i.e., 42.09% for the Simpson index, 39.66% for the Shannon index, and 79.75% for the Pielou index. Therefore, relationships between tree species diversity and elevation conformed to quadratic polynomial functions; the average amount of variation explained was 53.83%. With increasing elevation, unimodal change trends were observed, with an initial increase and then a decrease for the Simpson and Shannon indices (*P* > 0.05), whereas a significant increase was observed in the Pielou index (*P* < 0.01). This illustrated that tree species are mostly distributed at an intermediate elevation, where species are in balance and stable and thus develop a distribution pattern of higher species diversity.

## Discussion

### MDE hypothesis

Gradient features of species diversity of plant communities refer to regular changes in species diversity along a gradient of environmental factors at the community level^12,43^. The altitudinal gradient includes gradient effects of multiple environmental factors. It is therefore important to study altitudinal patterns of species diversity to reveal changes in biodiversity along environmental gradients. The width and range of species distribution along geographical gradients reflect the ecological adaptability, diffusivity, and evolutionary history of species^44^. To some extent, geographical distribution patterns of species diversity can be interpreted as outcomes of synthetic actions across altitudinal gradients resulting from eurychoric species with a greater distribution width and stenochoric species with a smaller distribution range along geographical gradients^44^. Hence, the MDE, environmental gradient, distribution area, human disturbance, and habitat heterogeneity all have effects on the vertical distribution patterns of species diversity^45,46^. According to the MDE hypothesis, there is overlap in the distribution range of species along altitudinal gradients; the highest overlap intensity occurs at intermediate elevations. There is relatively low overlap intensity at low and high elevation areas, and the peak values of species diversity occur at intermediate elevations.

In this study, forest ecosystems on the Loess Plateau were separated into three communities: tree, shrub, and herb communities. The altitudinal patterns and factors that influence species diversity of mountainous vegetation were then determined at the plant community level related to the form and family of the plant species. We discovered that the family numbers of the herb, shrub, and tree communities reached their greatest values at intermediate, intermediate, and lower elevations, respectively. We also discovered that correlations of species diversity indices with elevations conformed to unimodal change patterns for herb, shrub, and tree communities, which presented their greatest values at higher, lower, and intermediate elevations, respectively. This showed that MDE is another important factor that affects the distribution patterns of species diversity along regional altitudinal gradients, in addition to temperature, precipitation, and the terrain.

A large number of studies have already verified that MDE is a significant mechanism that influences the gradient patterns of species diversity. MDE not only functions along altitudinal gradients but also acts along latitudinal and temporal gradients^28–30,47^. However, the effects of MDE on species diversity patterns are highly controversial. Some studies considered MDE to be the main factor that results in unimodal patterns of species diversity^47,48^, whereas other studies affirmed that the effects of MDE are smaller in contrast to the functions of the distribution area, environmental gradient, and other factors^29,30^. Besides MDE, other factors may also lead to unimodal vertical gradient patterns of species diversity, such as plant productivity, human disturbance, and the regional climate^45,49^. Our research indicated that the relationships of species diversity conformed to unimodal change patterns along various elevations for mountainous herb, shrub, and tree communities in a semi-arid region of the temperate zone. It can be concluded that vertical patterns of species diversity with a unimodal type may be a more universal phenomenon, relative to monotonic decreasing or increasing patterns of species diversity with increasing elevations.

### Factors influencing MDE at the plant community level

In this study, more forbs and grasses were found at higher elevations, whereas more sedges occurred at lower elevations. The responses of the importance values of tree species to the altitudinal gradient demonstrated the following variation patterns: evergreen coniferous trees had higher importance values than deciduous coniferous trees, followed by deciduous broad-leaved trees. This showed that MDE was influenced by species life form. The species diversity of different life forms responded differently to the environment, and plant species with different life forms presented different diversity patterns along altitudinal gradients^50^. In New Zealand, the number of mountainous plants species decreased with increasing elevations and the total species number of all plants also decreased significantly, while species diversity had no significant distribution trend in response to elevation when plants with different life forms were considered under different layers of plant communities^51^.

MDE is a common pattern of species diversity of mountainous plants with changing elevations. Our study area, located on the Loess Plateau of China, belongs to a semi-arid mountainous region in the temperate zone where the maximum species diversity of the tree community occurred at intermediate elevations. This finding was in accordance with the MDE hypothesis. Research from the Kinabalu Mountains in Sabah, Malaysia, indicated an obvious MDE pattern of species number linked to elevations^52^. On the Haleakala Mountains, Hawaii, USA, the highest species diversity occurred at intermediate elevations, which was also in accordance with the MDE hypothesis^53^. The MDE hypothesis was also proved by studies conducted in the Yu Mountains of Taiwan and the Emei Mountains of Szechwan in China^54^. However, the MDE pattern of species diversity in tree communities is caused by precipitation, which is the highest at intermediate elevations^52^. This situation also occurred in the herb community.

There are many factors that affect the distribution of herbaceous plants, so the variations in species diversity in the herb community with elevations are complex^55^. In this study, we found that the herb community exhibited higher species diversity at higher elevations; more forbs and grasses were distributed at higher elevations, whereas more sedges were distributed at lower elevations. In the Siskiyou Mountains in Oregon, USA, the species diversity of herbaceous plants had a significantly positive correlation with elevation. This correlation occurred mainly due to an increase in the number of grass species, which was the primary reason that radiation was enhanced by a drastic reduction in community coverage as a result of increasing elevations. Consequently, there was an increase in the species diversity of herbaceous plants^24^. A decrease in species diversity with increasing elevations is a more familiar pattern for herbaceous plants in temperate^56^ and tropical^22^ forests.

We also discovered that the family numbers of herb and shrub species all showed unimodal change patterns with high values at their central elevations in this semi-arid region. This conformed to the MDE hypothesis as well. In arid temperate grasslands, species diversity indicated an MDE distribution pattern. For example, the species diversity of herbaceous plants presented an MDE pattern in drought areas of the Siskiyou Mountains^57^. However, in semi-humid mountains in the temperate zone, the species diversity of herbaceous plants was principally in control of the community structure, and community coverage did not respond uniformly to elevation. Studies in New Zealand showed that there were no evident distribution trends for species diversity of herbaceous plants along elevations^51^. In low bush communities of Chile, the species diversity of herbaceous plants declined with increasing elevations after longstanding succession, but it increased during the early stage of succession^58^. Therefore, relationships between the species diversity of herb species and elevations were not completely clear in semi-humid regions.

The major factors that control the distribution areas of species differ among different families and genera, and thus the vertical distribution patterns of species diversity differ^22^. We concluded that the family number of the tree species had a maximum distribution at lower elevations, unlike herb and shrub species; meanwhile, the responses of the importance values to the altitudinal gradient in the tree community were also different among evergreen coniferous trees, deciduous coniferous trees, and deciduous broad-leaved trees. These differences may have been related to environmental factors. Due to various distribution patterns of environmental parameters with elevations, the distribution patterns of species diversity showed large changes along elevations^59^. For example, the distribution of fern and Melastomataceae species is principally related to humidity, that of Acanthaceae and Bromeliaceae species is correlated with temperature, and that of Araceae species is related to transpiration^59^. Research conducted in the Gongga Mountains, China, showed that the species diversity with different floral components exhibited different distribution patterns along elevations due to differences in the environment and species origin^60^. We also discovered that the importance values of dominant families in the shrub (Rosaceae) and tree (Pinaceae) communities exhibited changing patterns in contrast to MDE. In our study, only the family numbers in the herb and shrub communities, as well as species diversity in the tree community, conformed to the MDE hypothesis. Therefore, we concluded that the MDE hypothesis of species diversity of mountainous vegetation is influenced by the species life form and family of different plant communities in the temperate semi-arid region of China.

### Factors influencing plant species diversity at the environment level

The altitudinal distribution patterns of the plant community diversity had greater discrepancies in mountainous regions and between different community types, which might be connected to regional environmental conditions, relative heights of mountains, and the geological landscape^35^. Concerning the altitudes of mountains, serious human disturbances (e.g., deforestation, grazing, and land-use conversion) had negative effects on biodiversity in low-altitude regions^61^. In high-altitude regions, the cold climate slowed down plant growth and soil development, while other harsh environments exceeded the tolerance limits for growth of the majority of species, such as by intense solar radiation or large temperature differences between day and night^62^. In the middle-altitude regions, the species diversity was relatively higher due to less human disturbances and the formation of transition zones of plant species differentiation between the low- and high-altitude regions^62^. Hence, the plant community diversity and its altitudinal gradient patterns in mountainous regions were largely influenced by regional climate and human disturbances.

Comparisons of the diversity at different levels indicated that the responses of the plant community diversity to the environment were not the same for diverse gradations, and different species exhibited different gradient patterns owing to restrictions from environmental factors^63,64^. The primary factors leading to the altitudinal differentiation of diversity included the temperature, moisture, soil nutrients, and succession process^65^. In our study area, compared with Guancen Mountain and Guandi Mountain, Wulu Mountain at the lower latitude of the Lvliang Mountains had a lower altitude and was located in the continental monsoon subhumid climate region of the warm temperate zone, making it suitable for the growth of secondary forests and shrub vegetation. However, the vegetation growth in the herb layer was restricted in Wulu Mountain, making diversity in the herb layer the greatest on Guandi Mountain at the middle latitude of the Lvliang Mountains^35^. This showed that the plant species diversity in the east of the Loess Plateau changed with the altitude, while being affected by complicated habitat conditions such as latitude and human disturbances. This characterization of the study area was correlated with the unimodal patterns observed. In this study, we observed that the family numbers of the herb and shrub communities presented unimodal patterns across an altitudinal gradient; the importance values of dominant families also presented unimodal patterns in the shrub and tree communities; and the species diversity indices of the herb, shrub, and tree communities conformed to unimodal change patterns following an altitudinal gradient as well.

In our recent studies on the species diversity of herbaceous communities in the Lvliang Mountains^66^, we found that the results calculated for β-diversity using different indices revealed the highest value for the Cody index and the lowest value for the Bray–Curtis index at altitudes between 1900 and 2000 m, indicating that areas located between 1900 and 2000 m form a transition zone in which the herbaceous community undergoes a rapid process of species renewal and changes in its composition. The results for *γ*-diversity indicated a pattern of unimodal variation in relation to altitude. Changes in altitude gradient had highly significant impacts on changes in temperature and humidity, indicating that various environmental factors (notably humidity and temperature) and human disturbances had combined effects on changes in the values of the *α*-diversity indices.

At present, it is widely believed that the formation of herbs in different life forms was principally impacted by precipitation, whereas in areas with similar rainfall, water, heat, and light conditions need to be considered. These conditions chiefly included average annual precipitation, accumulated temperature, and illumination time^67^. In this research, we observed that herb and tree species in different life forms showed different trends with altitudinal gradients in the Lvliang Mountains. At higher elevations, forbs and grasses grew well, whereas sedges grew well at lower elevations. The responses of different tree life forms to the altitudinal gradient were greater for evergreen coniferous tree species than for deciduous coniferous tree species and deciduous broad-leaved tree species. From the north to the south in the Lvliang Mountains, increases in the average annual precipitation increased the number of species and components of the annual herbs, while the hydrothermal matching requirements of Guandi Mountain at the middle latitude were preferred for annual herb growth in comparison with Guancen Mountain at the higher latitude^67^. However, considering whole mountains, the Lvliang Mountains located in the continental monsoon climate region of the warm temperate zone had four distinctive seasons with drought and wind in the spring, and a quick rise in air temperatures, and larger diurnal temperature differences^35^. These conditions conformed to the habitat features of herbs and trees. Hence, the hydrothermal distribution status affected by latitude and human disturbances determined the altitudinal distribution patterns of plant community diversity in the Lvliang Mountains.

### The MDE at different elevations

In this study, we discussed the MDE of mountainous vegetation on the Loess Plateau with an elevational range from 1324 to 2745 m, including tree, shrub and herb community. This range was a very large elevation range for a case study, but a very short range in comparison to global elevation ranges, which extended from the sea level to well over 8000 m (though the highest locations did not have any vegetation). Therefore, owing to this limitation, the results we obtained in this research were suitable for lower elevation mountains in semi-arid areas.

The MDE was changed with different elevations and vegetation layers. In studies on the Daiyun Mountains with an elevation from 900 to 1600 m, the phylogenetic diversity and species diversity of tree community indicated an intermediate high expansion pattern along elevations and their peak values all appeared at the elevation of 1200 m^68^. This conclusion conformed to the MDE pattern. In our studies on the Lvliang Mountains with an elevation from 1459 to 2610 m, higher species diversity of tree community was observed at intermediate elevations with a peak value being at the 2000 m, which conformed to the MDE pattern either. Therefore, at smaller elevations less than 2600 m, species diversity of tree conformed to the MDE pattern.

When an elevation reached 2700 m on the Lvliang Mountains, the vegetation types changed to shrub and grass, and only their family numbers followed the MDE pattern across an altitudinal gradient. Slimily, the species richness of shrub and herb layer showed an obvious “lateral pattern” on an elevational gradient from 2950 to 3750 m on the Three River Headwater, both reaching the maximum value at the 3150 m; while with the rise of altitude, α diversity of shrub layer and herb layer showed a “wave”-shaped changed trend, reaching the lowest value at the 3550 m^69^. It illustrated that species diversity of shrub and grass did not conform to the MDE pattern completely at medium elevations from 2700 to 3700 m.

As for an elevation extending from 3000 to 4400 m on the Gongga Mountains, the vegetation type was alpine meadow, and the species richness index presented an obvious unimodal pattern with a peak value at the 3850 m, which accorded with the MDE pattern^70^. Similarly, studies on an alpine meadow on the Gannan revealed that the number of richness, Shannon-Weiner index and phylogenetic diversity of plant community all showed a “humped-back” relationship with the increase of altitude from 3000 to 4000 m, and reached the maximum value at the 3800 m^71,72^. Thereby, at greater elevations more than 3800 m, species diversity of alpine meadow conformed to the MDE pattern.

However, when an elevation exceeded 5000 m, research object on species diversity were not vegetation but animals along an altitudinal gradient. For example, on the Himalaya Mountains with an elevation from 3755 to 5016 m, the ant species richness illustrated a “unimodal curve” pattern along the rise of altitude, and the Shannon–Wiener index and Fisher α index of ant community commonly expressed the “Multi-Domain Effect” phenomenon^73^. Another research on mammalian richness was also conducted on the Himalaya Mountains. It concluded that most of elevational species richness patterns were hump-shaped from 100 to 6000 m on the Himalayas Mountains^74^. As a result, the MDE pattern was also extremely common in animal communities.

## Conclusions

According to species life form and family flora of various plant communities, the MDE hypothesis of mountainous vegetation was verified by the altitudinal patterns of species diversity along different elevations in the east of the Loess Plateau, China.

1. The family number of the herb community, with Cyperaceae as the dominant family, followed the MDE pattern across altitudinal gradients, but there was greater species diversity at higher elevations, which did not conform to the MDE hypothesis. Forbs and grasses occurred mostly at higher elevations, whereas sedges occurred mostly at lower elevations.
2. The family number of the shrub community, with Rosaceae as the dominant family, also exhibited the MDE pattern across an altitudinal gradient, but the importance value of the dominant family had the opposite pattern from MDE with elevations.
3. The family number and importance value of the tree community, with Pinaceae as the dominant family, did not present MDE patterns along variations in an altitudinal gradient. However, higher species diversity was observed at intermediate elevations, which conformed to the MDE hypothesis. The responses of the importance values of different species demonstrated variation patterns that were greater for evergreen coniferous trees than for deciduous coniferous and deciduous broad-leaved trees.

As a whole, the family number of the herb and shrub communities as well as the species diversity of the tree community were in accordance with the MDE hypothesis, indicating that the MDE pattern of species diversity is influenced by community life form and the family flora of mountainous vegetation. However, plant community diversity and its altitudinal gradient patterns were largely influenced by regional climate and human disturbances in mountainous regions, which may have caused the deviation from the MDE hypothesis. The plant species diversity in the Lvliang Mountains changed with the altitude, while being affected by complicated habitat conditions, such as the latitude and human disturbances. This characterization of the study area was correlated with the unimodal patterns observed. Hence, the hydrothermal distribution status affected by latitude and human disturbances determined the altitudinal distribution patterns of plant community diversity on the Loess Plateau of China.

## Supplementary Information

Supplementary Information.

## Data Availability

All relevant data are within the manuscript and its Supplementary Information files.
