# Supplementary material for: The mid-domain effect of mountainous plants is determined by community life form and family flora on the Loess Plateau of China
Source: Sci Rep. 2021 May 26;11:10974. doi: 10.1038/s41598-021-90561-4 (PMC8155023; doi:10.1038/s41598-021-90561-4)
Supplement: Supplementary file 1 — Supplementary Information. [file 41598_2021_90561_MOESM1_ESM.docx]

**Title page**

**The mid-domain effect of mountainous plants is determined by community life form and family flora on the Loess Plateau of China**

Manhou Xu ^a, b, *^, Rong Du ^a^, Xiaoli Li ^a^, Xiaohui Yang ^a^, Baogui Zhang ^a^, Xiuli Yu ^a^

^a^ Institute of Geographical Science, Taiyuan Normal University, Jinzhong 030619, China

^b^ Key Laboratory of Restoration Ecology of Cold Area in Qinghai Province, Northwest Institute of Plateau Biology, Chinese Academy of Sciences, Xining, 810008, China

***** xumh@tynu.edu.cn

**Supplementary Table**

S1. Species name, height, coverage, density, and frequency of herb, shrub, and tree in all surveyed plots. Plant species frequency was not calculated in shrub and tree communities.

| **Life form** | **Species** | **Height (m)** | **Coverage (%)** | **Density (plant/m^2^)** | **Frequency (%)** |
| --- | --- | --- | --- | --- | --- |
| Herb | *Kobresia humilis* | 0.01 | 8.60 | 209.33 | 37.60 |
|  | *Artemisia argyi* | 0.02 | 23.20 | 344.00 | 40.00 |
|  | *Pulsatilla chinensis* | 0.01 | <0.01 | 0.20 | 0.10 |
|  | *Pinellia ternata* | 0.02 | 1.40 | 26.66 | 7.10 |
|  | *Sanicula chinensis* | 0.03 | 0.60 | 10.60 | 1.30 |
|  | *Scutellaria scordifolia* | 0.02 | 0.50 | 10.50 | 1.30 |
|  | *Astragalus scaberrimus* | <0.01 | <0.01 | 0.80 | 0.20 |
|  | *Plantago asiatica* | 0.01 | 2.20 | 12.69 | 7.50 |
|  | *Cirsium setosum* | 0.01 | 0.50 | 0.88 | 0.60 |
|  | *Lathyrus davidii* | 0.01 | 0.10 | 2.10 | 0.40 |
|  | *Bupleurum longiradiatum* | 0.02 | 0.60 | 1.14 | 0.90 |
|  | *Spodiopogon sibiricus* | 0.02 | 0.10 | 0.86 | 0.90 |
|  | *Speranskia tuberculata* | 0.01 | 0.10 | 0.80 | 0.10 |
|  | *Sanguisorba officinalis* | 0.02 | 0.60 | 4.86 | 2.90 |
|  | *Heracleum hemsleyanum* | 0.01 | <0.01 | 0.20 | 0.10 |
|  | *Aconitum polyschistum* | <0.01 | 0.10 | 0.27 | 0.10 |
|  | *Cynanchum chinense* | 0.02 | 10.70 | 122.67 | 49.30 |
|  | *Potentilla bifurca* | <0.01 | 0.30 | 2.67 | 2.70 |
|  | *Platanthera chlorantha* | <0.01 | 0.10 | 0.80 | 0.60 |
|  | *Saussurea japonica* | 0.02 | 2.30 | 10.80 | 9.60 |
|  | *Stachys affinis* | 0.03 | 1.70 | 2.57 | 2.30 |
|  | *Heteropappus hispidus* | 0.10 | 1.30 | 30.93 | 3.20 |
|  | *Rorippa indica* | 0.02 | 1.20 | 6.32 | 1.90 |
|  | *Actinostemma tenerum* | 0.01 | <0.01 | 0.40 | 0.10 |
|  | *Lappula myosotis* | <0.01 | 0.10 | 0.57 | 0.30 |
|  | *Ainsliaea elegans* | 0.02 | 1.20 | 3.23 | 2.30 |
|  | *Lycianthes biflora* | 0.01 | 0.20 | 1.00 | 0.20 |
|  | *Saxifraga stolonifera* | 0.02 | 0.50 | 10.40 | 1.90 |
|  | *Polemonium coeruleum* | 0.02 | 0.70 | 1.79 | 1.30 |
|  | *Scutellaria baicalensis* | 0.01 | <0.01 | 0.20 | 0.10 |
|  | *Leontopodium leontopodioides* | 0.02 | 2.20 | 53.24 | 11.70 |
|  | *Sedum stellariifolium* | 0.02 | 0.40 | 2.29 | 1.70 |
|  | *Equisetum ramosissimum* | 0.02 | 0.30 | 1.20 | 1.20 |
|  | *Rubia membranacea* | 0.03 | 2.30 | 3.00 | 3.00 |
|  | *Viola verecunda* | 0.03 | 0.20 | 1.60 | 0.50 |
|  | *Cerastium arvense* | 0.01 | 1.70 | 27.52 | 6.90 |
|  | *Sphaerophysa salsula* | 0.16 | 1.80 | 21.07 | 4.30 |
|  | *Graptopetalum pachyphyllum* | 0.01 | <0.01 | 0.60 | 0.20 |
|  | *Oxytropis coerulea* | 0.01 | 1.70 | 10.95 | 6.70 |
|  | *Geranium wilfordii* | 0.02 | 0.30 | 3.38 | 1.00 |
|  | *Androsace henryi* | 0.01 | 0.10 | 12.80 | 0.30 |
|  | *Czernaevia laevigata* | 0.01 | 0.10 | 0.70 | 0.20 |
|  | *Agrimonia pilosa* | 0.04 | 0.20 | 1.10 | 0.30 |
|  | *Geum aleppicum* | 0.04 | 0.40 | 2.54 | 0.60 |
|  | *Pedicularis shansiensis* | 0.02 | 1.80 | 12.93 | 7.70 |
|  | *Ranunculus japonicus* | 0.03 | 0.10 | 0.20 | 0.10 |
|  | *Dracocephalum rupestre* | <0.01 | 0.30 | 0.86 | 0.60 |
|  | *Potentilla fragarioides* | 0.02 | 8.60 | 68.00 | 37.20 |
|  | *Artemisia eriopoda* | 0.01 | 0.10 | 1.40 | 0.40 |
|  | *Patrinia villosa* | 0.01 | 0.10 | 0.80 | 0.20 |
|  | *Elymus dahuricus* | 0.02 | 1.30 | 59.93 | 17.40 |
|  | *Taraxacum mongolicum* | 0.03 | 7.70 | 33.33 | 25.30 |
|  | *Rhaponticum uniflorum* | 0.01 | 0.10 | 0.20 | 0.10 |
|  | *Rubia cordifolia* | 0.01 | 0.20 | 0.80 | 0.20 |
|  | *Gentiana macrophylla* | 0.01 | 0.70 | 0.86 | 0.90 |
|  | *Artemisia carvifolia* | 0.02 | 0.90 | 7.20 | 4.60 |
|  | *Avena sativa* | 0.03 | 0.60 | 7.71 | 5.40 |
|  | *Blumea mollis* | 0.02 | 0.10 | 3.00 | 0.60 |
|  | *Adenophora stricta* | <0.01 | 0.10 | 0.86 | 0.60 |
|  | *Allium senescens* | 0.03 | 0.10 | 2.13 | 0.30 |
|  | *Lathyrus quinquenervius* | 0.01 | 0.60 | 1.83 | 1.60 |
|  | *Ostericum sieboldii* | 0.01 | 0.10 | 1.60 | 0.30 |
|  | *Galium bungei* | 0.04 | 0.90 | 9.63 | 4.40 |
|  | *Rumex acetosa* | 0.01 | 0.10 | 0.33 | 0.20 |
|  | *Carex tristachya* | 0.12 | 10.40 | 332.92 | 30.70 |
|  | *Thalictrum aquilegifolium* | 0.01 | 4.10 | 60.39 | 19.60 |
|  | *Ligularia fischeri* | 0.02 | 0.20 | 1.51 | 0.70 |
|  | *Heteropappus altaicus* | 0.16 | 3.80 | 55.00 | 7.10 |
|  | *Clematis hexapetala* | 0.06 | 1.30 | 14.67 | 2.30 |
|  | *Chrysanthemum coronarium* | 0.01 | 0.60 | 1.14 | 0.90 |
|  | *Vicia unijuga* | 0.03 | 0.30 | 2.88 | 1.00 |
|  | *Potentilla chinensis* | 0.06 | 0.30 | 2.77 | 0.80 |
|  | *Aconitum carmichaeli* | 0.02 | 0.50 | 3.01 | 1.10 |
|  | *Galium linearifolium* | 0.01 | 0.20 | 3.60 | 0.50 |
|  | *Kobresia capillifolia* | 0.06 | 13.50 | 405.60 | 56.00 |
|  | *Potentilla longifolia* | 0.02 | 1.60 | 15.32 | 10.20 |
|  | *Valeriana officinalis* | 0.05 | 0.10 | 0.86 | 0.90 |
|  | *Rubus reflexus* | 0.02 | 0.20 | 1.80 | 0.50 |
|  | *Carpesium cernuum* | 0.02 | 0.20 | 1.20 | 0.30 |
|  | *Festuca ovina* | 0.02 | <0.01 | 3.27 | 0.50 |
|  | *Fragaria vesca* | 0.04 | 9.20 | 145.31 | 18.40 |
|  | *Allium ramosum* | 0.04 | 0.10 | 1.00 | 0.30 |
|  | *Deyeuxia arundinacea* | 0.02 | 0.10 | 4.76 | 0.40 |
|  | *Vicia sepium* | 0.02 | 0.10 | 0.63 | 0.20 |
|  | *Siphonostegia chinensis* | 0.09 | 0.60 | 5.67 | 1.30 |
|  | *Polygala tenuifolia* | 0.01 | 0.10 | 1.03 | 0.20 |
|  | *Viola prionantha* | 0.01 | 0.40 | 14.40 | 1.60 |
|  | *Poa annua* | 0.04 | 0.30 | 4.53 | 1.20 |
|  | *Stipa capillata* | 0.02 | 3.30 | 220.00 | 16.00 |
|  | *Eragrostis ferruginea* | 0.04 | 1.40 | 85.40 | 3.10 |
|  | *Lespedeza chinensis* | 0.02 | 0.10 | 2.13 | 0.90 |
|  | *Polygonum viviparum* | 0.02 | 14.80 | 216.74 | 42.10 |
|  | *Viola philippica* | 0.01 | 0.10 | 0.27 | 0.10 |
|  | *Aster tataricus* | 0.01 | 0.40 | 1.60 | 1.10 |
| Shrub | *Rosa xanthina* | 1.78 | 13.70 | 0.04 | — |
|  | *Rosa hugonis* | 1.52 | 5.70 | 0.03 | — |
|  | *Daphne giraldii* | 0.70 | 0.10 | <0.01 | — |
|  | *Cotoneaster acutifolia* | 0.86 | 0.30 | <0.01 | — |
|  | *Potentilla fruticosa* | 0.31 | 0.70 | 0.05 | — |
|  | *Hippophae rhamnoides* | 0.74 | 14.90 | 0.17 | — |
|  | *Rosa davurica* | 3.38 | 21.20 | 0.01 | — |
|  | *Cotoneaster multiflorus* | 2.70 | 0.90 | <0.01 | — |
|  | *Spiraea pubescens* | 0.82 | 0.50 | 0.02 | — |
|  | *Pyracantha fortuneana* | 9.20 | 15.20 | 0.01 | — |
|  | *Potentilla glabra* | 0.51 | 0.90 | 0.02 | — |
| Tree | *Betula platyphylla* | 7.41 | 24.50 | 0.03 | — |
|  | *Populus tomentosa* | 8.06 | 50.90 | 0.03 | — |
|  | *Platycladus orientalis* | 4.25 | 4.90 | 0.01 | — |
|  | *Salix matsudana* | 6.50 | 18.10 | <0.01 | — |
|  | *Betula albo-sinensis* | 8.01 | 32.90 | 0.02 | — |
|  | *Larix principis-rupprechtii* | 14.48 | 42.60 | 0.02 | — |
|  | *Quercus liaotungensis* | 7.55 | 33.60 | 0.02 | — |
|  | *Koelreuteria paniculata* | 7.44 | 32.40 | 0.01 | — |
|  | *Acer buergerianum* | 4.17 | 23.10 | 0.01 | — |
|  | *Crataegus pinnatifida* | 2.73 | 7.50 | 0.01 | — |
|  | *Salix pseudotangii* | 2.48 | 8.70 | 0.01 | — |
|  | *Populus davidiana* | 8.54 | 29.70 | 0.01 | — |
|  | *Populus simonii* | 11.29 | 210.70 | 0.02 | — |
|  | *Pinus tabuliformis* | 9.49 | 112.80 | 0.07 | — |
|  | *Picea asperata* | 11.12 | 117.60 | 0.06 | — |
